# Bibliographical Notices

**Published:** 1844-11-16

**Authors:** 


### BIBLIOGRAPHICAL NOTICES.

*Reply of* William A. McDowell *to* Dr. Yandell's *Rejoinder, in a controversy relative to the cure of Consumption.* Louisville, Ky. 1844.

This is a pamphlet of thirty pages, in which the author endeavours to sustain himself against the arguments and allegations contained in the Western Journal of Medicine for October last. We have been too much disgusted with advertisements put forth in newspapers, pamphlets, and every other way best calculated to catch the eye of the suffering and credulous, announcing "*consumption curable*," &c., not to feel suspicious of every thing that bears that stamp. Nevertheless, Dr. McDowell, as well as every one else, has a perfect right to entertain such an opinion, and to promulgate it, provided it is done in good faith and with honest intentions. But to entitle any one to claim such an acknowledgement of motives, it is indispensable, in our estimation, that he shall "*give a reason for the faith that is in him.*" Let us have a statement, not alone of *his experience*, but of his views of the pathology of the disease and the means he employs in the treatment. We can then judge of the correspondence of his observations with those of others, the consistency, safety, and rational adaptation of the means to the end or object to be fulfilled.

The present publication is not of that character. It relates chiefly to facts in dispute between the writers, about which those at a distance can know nothing, and will hardly feel much interest. The parade of cases alleged to have been cured, but without mention of the means, which constitutes a large portion of the publication, is little likely to impress professional men favourably towards the writer.

---

### SYDENHAM SOCIETY'S WORKS.

THOMÆ SYDENHAM, M. D. *Opera Omnia.* Editit GULIELMUS ALEXANDER GREENHILL, M. D. 8vo. pp. 668. Londini. 1844.

The last and the most beautiful volume of the works published by the Sydenham Society, for the year 1843, has reached the subscribers in this country. It, alone, is richly worth the price subscribed for the three volumes.

This complete edition of the works of the great master, is printed from the *opera universa*, published by Walter Kettilby in 1685; and it contains, in addition, the *Schedula monitoria de novæ febris ingressu*—Kettilby's second edition, Lond. 1688;—and the *Processus Integri in morbis ferè omnibus curandis*, A. D. Thoma Sydenham, M. D., conscripti, &c. &c.; necnon *De Phthisi Tractatulo*, &c. &c. Lond. 1695.

The Editor, Dr. Greenhill, has carefully revised the original Latin; yet, from the difficulty of printing works, in the learned languages, even in England, his table of errata is by no means meagre. We have stumbled on one error that has escaped him, and there must inevitably be others. In the formula, for example, at the top of page 560, *Albumen* occurs in the nominative, instead of in the genitive case. Dr. Greenhill has added brief notes with the view of illustration or explanation; has rendered the index to the Leipsic edition shorter, but equally satisfactory; and has appended an index of proper names; and another of medical formulæ, and of the articles occurring in the formulæ; giving, at the same time, the modern names for the different articles of the materia medica.

"Faxit DEUS"—inquit Editor "ter Opt. Max. ut et hic et omnes nostræ vocationis labores, itemque omnia a nova hac Societate Sydenhamiana suscepta, cedant in IPSIUS laudem et hominum emolumentum;"—to which we cordially say, AMEN! Thus far, certainly, the action of the Society has been most favourable to all concerned; and we fervently hope that they may not relax in their useful exertions.

